# Preclinical Absorption, Distribution, Metabolism, and Excretion of an Oral Amide Prodrug of Gemcitabine Designed to Deliver Prolonged Systemic Exposure

**DOI:** 10.3390/pharmaceutics5020261

**Published:** 2013-05-08

**Authors:** Enaksha Wickremsinhe, Jingqi Bao, Richard Smith, Richard Burton, Shannon Dow, Everett Perkins

**Affiliations:** 1 Drug Disposition, Eli Lilly and Company, Indianapolis, IN 46278, USA; E-Mails: bao_jing_q@lilly.com (J.B.); richard.smith@quintiles.com (R.S.); richard.burton@quintiles.com (R.B.); enaksha@lilly.com (S.D.); perkins.e@lilly.com (E.P.); 2 Current affiliation: Quintiles, Inc., Plainfield, IN 46241, USA

**Keywords:** gemcitabine, prodrug, carboxylesterase, CES2

## Abstract

Gemcitabine is an intravenously administered nucleoside analog chemotherapeutic agent. The ability to deliver this agent as an oral drug would allow greater flexibility of administration and patient convenience; however, attempts have been fraught with high first-pass metabolism and potential intestinal toxicity. Alternatively, an amide prodrug of gemcitabine (LY2334737) was discovered, which is able to avoid the extensive first-pass metabolism that occurs following administration of gemcitabine. Preclinical *in vitro* and *in vivo* experiments were conducted to evaluate the hydrolysis and pharmacokinetics of LY2334737 and its downstream metabolites. In mice, rats, and dogs, the prodrug is absorbed largely intact across the intestinal epithelium and delivers LY2334737 to systemic circulation. The hydrolysis of LY2334737 is relatively slow, resulting in sustained release of gemcitabine *in vivo*. *In vitro* experiments identified carboxylesterase 2 (CES2) as a major enzyme involved in the hydrolysis of LY2334737, but with relatively low intrinsic clearance. Following hydrolysis of the prodrug, gemcitabine is cleared predominantly via the formation of its inactive metabolite dFdU. Both biliary and renal excretion was responsible for the elimination of LY2334737 and its metabolites in both mice and dogs.

## 1. Introduction

Gemcitabine (Gemzar^®^, 2',2'-difluoro-deoxycytidine, dFdC) is a close structural analog of the nucleoside deoxycytidine and has a broad spectrum of anti-tumor activity against several human malignancies including pancreatic [1,2], ovarian [3,4], lung [5,6], breast [7,8], and bladder [9,10]. It is administered intravenously (IV) in the clinic as a 30 min infusion on days 1 and 8 of each 21-day cycle or days 1, 8, and 15 of each 28-day cycle [11]. Gemcitabine is phosphorylated intracellularly by deoxycytidine kinase to gemcitabine diphosphate (dFdCDP) and gemcitabine triphosphate (dFdCTP), which gets incorporated into DNA leading to apoptosis [12,13,14,15]. The cytotoxicity of gemcitabine is further enhanced by the inhibition of ribonucleotide reductase by dFdCDP, resulting in a reduction in the intracellular deoxycytidine triphosphate pools and an increase in the incorporation of dFdCTP into DNA [14,16]. The clearance of gemcitabine is driven by rapid and extensive inactivation by cytidine deaminase, which is present ubiquitously and at high levels in both plasma and in the liver, to its primary metabolite, 2',2'-difluoro-deoxyuridine (dFdU) [14,17].

Clinical studies have shown that efficacy can be achieved at lower plasma gemcitabine concentrations, along with reduced toxicity, when accompanied with longer infusion times [18,19]. Increased survival time with improved quality of life was reported compared to the standard dosing regimen in a Phase II study where gemcitabine was infused over 24 h at a 10-fold lower dose (100 mg/m^2^) to patients with advanced pancreatic adenocarcinoma [20]. Collectively, these clinical studies indicate that the anti-tumor effect of gemcitabine is schedule dependent and that lower doses are efficacious. Therefore, it could be advantageous to deliver gemcitabine in a manner where it can achieve prolonged systemic exposure, good efficacy with lower toxicity along with added flexibility of administration and greater patient convenience, *i.e.*, an oral formulation. However, administering gemcitabine orally to patients has been fraught with limitations including low oral bioavailability, high first-pass clearance, variable systemic exposures during dose escalation studies, and observation of GI toxicity including nausea, vomiting, and diarrhea [21].

A possible option that could overcome these challenges is a prodrug approach that can safely deliver gemcitabine with minimal intestinal activation, have good bioavailability, and low hepatic activation. Prodrug strategies are utilized during drug development to improve physiochemical, biopharmaceutical or pharmacokinetic properties of the molecule. They are typically active drug entities that have been chemically modified by covalently binding a judiciously selected chemical group that would render it pharmacologically inactive and would be converted to the active agent following metabolic biotransformation. The pro-moiety can often govern the physio-chemical properties of the molecule including the solubility of the drug, its stability, the rate at which it liberates the active drug moiety and the particular enzyme(s) required for its biotransformation [22].

Prodrugs have proven to be an effective method for the oral delivery of several nucleoside drugs such capecitabine and tegafur-uracil (5-fluorouracil, 5-FU), valacyclovir (acyclovir), valganciclovir (ganciclovir) [22,23]. These prodrugs enhance oral absorption of their parent molecules and/or decrease toxicity due to the parent molecule. Oral prodrugs of 5-FU, such as capecitabine and uracil, have been developed in order to mimic the protracted infusion schedule of 5-FU while offering the convenience of an orally-administered therapy with potentially fewer toxic effects (and reduced administration costs) [22,24].

LY2334737 is an amide prodrug of gemcitabine that was developed to be absorbed intact and cleaved *in vivo*, releasing gemcitabine and valproic acid [25]. Amide prodrugs, although less commonly used during drug development possibly due to their high enzymatic stability *in vivo* [26], are usually hydrolysed by ubiquitous carboxylesterases, peptidases or proteases to release the active drug moiety [27,28]. Early preclinical and *in vitro* data have shown that LY2334737 is more stable to both enzymatic and chemical hydrolysis and leads to improved bioavailability by blocking the site of deamination to its uridine metabolite, thus reducing its first-pass metabolism [25]. This can result in prolonged systemic exposure of gemcitabine compared to both IV and oral administration of gemcitabine.

The current studies were conducted to evaluate the pharmacokinetics, metabolism, and excretion of an amide prodrug of gemcitabine that was designed to be dosed orally.

## 2. Experimental Section

### 2.1. Test Material

**Figure 1:**
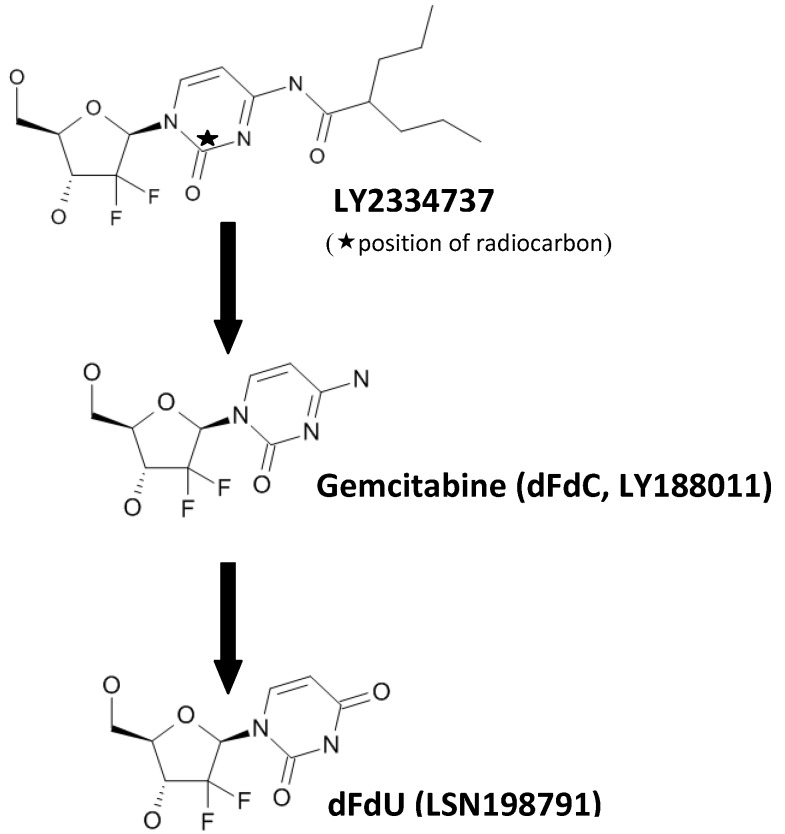
Structures of LY2334737, gemcitabine and dFdU.

LY2334737 (2'-deoxy-2',2'-difluoro-*N*-(1-*oxo*-2-propylpentyl)-cytidine) hemi-*p*-toluenesulfonic acid hemihydrate (potency 79.2%), [14C] LY2334737 hemi-*p*-toluenesulfonic acid hemihydrate (radiochemical purity 99.02%) were obtained from Lilly Research Laboratories (Eli Lilly and Co., Indianapolis, IN, USA). Molecular weights of LY2334737, gemcitabine, and dFdU are 389.4, 263.2, and 264.2, respectively (Figure 1).

### 2.2. *In Vitro* Studies

Mouse, dog, monkey, and human small intestine homogenates (SIH) and liver S9 preparations were prepared in 50 mM ammonium acetate buffer (pH = 7.4) with 1 mM CaCl_2_ and 1 mM MgCl_2_ at 5 mg/mL and 2 mg/mL protein concentrations, respectively. SIH preparations were incubated with 100 µM LY2334737 while S9 preparations were incubated with 10 µM LY2334737 in the presence of NADPH, at 37 °C for 6 h. Replicates of each were removed for analysis at 0, 0.5, 2, and 6 h incubation. Enzyme kinetic experiments with SIH and S9 fractions were conducted with a 2 mg/mL protein concentration using LY2334737 concentrations ranging from 10 to 800 µM and incubated at 37 °C for 2 h. Tetrahydrouridine (THU) was used during all incubations to inhibit cytidine deaminase. Purified carboxyl esterase 1A1 (CES1A1) and carboxyl esterase 2 (CES2) enzymes were obtained from Lilly Research Laboratories (Eli Lilly and Co., Indianapolis, IN) and incubated with LY2334737 at 37 °C for 2 h with 1, 10, and 100 µg/mL of enzyme. Aliquots of 100 µL were mixed with 125 µL acetonitrile and 25 µL of gemcitabine internal standard (20 µg/mL), centrifuged, and the supernatant was analyzed by LC-MS to quantify the amount of gemcitabine released. A Waters ZX mass spectrometer (Waters, Milford, MA, USA) and a Waters 2690 HPLC system (Waters, Milford, MA, USA) was used with a Zorbax SB-CN 4.6 × 250 mm, 5 µm HPLC column (Agilent, Santa Clara, CA, USA) using a 50 mM ammonium acetate and acetonitrile mobile phase each containing 0.12% formic acid.

### 2.3. Pharmacokinetic Studies

CD-1 mice, Beagle dogs and Sprague-Dawley rats were dosed with a single oral administration of LY2334737 as the hemi-*p*-toluenesulfonic acid hemi-hydrate formulated in purified water with 5% ethanol. Mice and dogs were also dosed with a single oral dose of gemcitabine formulated in purified water. Plasma samples were collected into tubes containing heparin and tetrahydrouridine (THU) starting from 0.5 h through 24 h postdose. All samples were stored at −70 °C.

### 2.4. Radiolabeled Studies

Radiolabeled gemcitabine prodrug, ^14^C-LY2334737 (Figure 1) was dosed to CD-1 mice as a single 10 mg/kg oral dose (100 µCi/kg) in purified water and to Beagle dogs as a single 5 mg/kg (30 µCi/kg) oral gavage (formulated in 5% ethanol in purified water) or IV bolus (formulated in 5% ethanol in saline). All animals were fasted overnight and 2 h postdose. *Mouse blood collection*: Blood samples were collected via cardiac puncture (following anesthetizing with carbon dioxide) into tubes containing heparin and THU at 0.5, 1, 2, 4, 6, 8, 12, 24, 36, and 48 h postdose (4 mice per time point; 1 sample per mouse, and pooled). Additional mice were used to collect plasma at 0.5, 2, 8, and 24 h postdose (4 mice per time point; 1 sample per mouse and pooled) for metabolite identification. Approximately 0.25 mL blood was removed for determination of radioactivity. Remaining blood was centrifuged to obtain plasma for bioanalysis and determination of plasma radioactivity. *Dog blood collection*: Blood samples (approximately 3 mL) were collected via the jugular vein into tubes containing heparin and THU. Samples were collected at 0.5, 1, 2, 4, 8, 12, 24, 36, and 48 h postdose following oral and at 0.8, 0.25, 0.5, 1, 2, 4, 8, 12, 24, 36, and 48 h postdose following IV dosing. Approximately 0.5 mL was removed for determination of radioactivity. Remaining blood was centrifuged to obtain plasma for bioanalysis, determination of plasma radioactivity, and metabolite identification. *Excretion samples*: Mouse and dog urine samples were collected from 0 to 6 h, 6 to 24 h, and at every 24 h interval post dose. Feces and cage wash samples were collected every 24 h. Cage wash was collected by rinsing the cage with 50 mL of 1:1 methanol:water (*v*:*v*), followed by a deionized water rinse. Fecal samples were mixed with a 1:1 methanol:water (*v*:*v*) solution (1–2 mL/g feces), soaked, then shaken for 4 h, mixed using a polytron to produce a homogeneous slurry. Mice were euthanized by asphyxiation with CO_2_ followed by cervical dislocation and the carcass was collected at 168 h (study termination). All samples were stored at −70 °C prior to analysis.

### 2.5. Plasma Extraction and LC-MS/MS Analysis

LY2334737, gemcitabine (dFdC, LY188011), dFdU and corresponding stable-isotope labeled internal standards (IS) were extracted by solid phase extraction using Oasis HLB 30 mg solid phase extraction plates (Waters Inc., Milford, MA, USA). Final elution (in methanol) was evaporated to dryness under nitrogen, reconstituted with water and separated on a Betasil C18, 2.1 × 100 mm, 5 µm (Thermo Electron Corp. West Palm Beach, FL, USA) HPLC column. LY2334737 was quantified using isocratic conditions while gemcitabine and dFdU were quantified using gradient elution. Mobile phase consisted of water and acetonitrile, each containing 0.02% trifluoroacetic acid at a 0.5 mL/min flow rate and ambient column temperature. Mass spectrometric data were generated using an API 4000 triple quadrupole mass spectrometer and acquired using Analyst Software, v1.4 (Applied BioSystems, Foster City, CA, USA). Selected reaction monitoring (SRM) was performed using the heated nebulizer inteface operated at 450 °C in positive ion mode using a nebulizer setting at 3. Optimized SRM transitions for LY2334737 (390.1→112.0), gemcitabine (264.1→112.0) and dFdU (265.1→113.0) and its corresponding stable labeled internal standards were used for quantification using Analyst v1.4 (Applied BioSystems, Foster City, CA, USA).

### 2.6. Analysis of Radioequivalents

*Blood*: Approximately 0.2 g aliquots were allowed to dry and combusted in a Perkin-Elmer Oxidizer Model 307 (Perkin-Elmer, Waltham, MA, USA). The ^14^CO_2_ was trapped and assayed for radioactivity using a Perkin-Elmer tricarb liquid scintillation counter (LSC, Perkin-Elmer, Waltham, MA, USA). The minimum detectable activity (MDA) for blood was 13 DPM. *Plasma, urine and cage wash*: Approximately 0.2 g aliquots were mixed with 12 mL of Beckman Ready Protein Plus℘scintillation counting solution (Beckman Coulter Inc., Brea, CA, USA) and assayed by LSC. The MDA for plasma, urine, and cage wash were was 7, 8, and 7 DPM, respectively. *Feces*: Approximately 0.5 g aliquots were allowed to dry overnight, combusted and analyzed similar to blood—described above. The MDA for feces was 12 DPM. *Carcasses*: Were gently boiled in 150 mL of ethanol and 25 g of potassium hydroxide until all tissues dissolved. Approximately 0.2 g aliquots were mixed with 12 mL Beckman Ready Protein Plus℘ and 0.2 mL of acetic acid and assayed by LSC. The MDA for carcass was 8 DPM.

### 2.7. Sample Preparation for Metabolite Identification

Plasma samples (approximately 250 µL) were extracted twice with 1 mL aliquots of denatured ethanol and combined, evaporated to dryness under N_2_ at 49 °C and reconstituted in 300 µL 0.2% formic acid/methanol (95:5, *v*:*v*). Urine samples were diluted with 0.2% formic acid and analyzed without additional sample processing. Feces samples (0.4 to 0.6 g) were extracted with 3 mL methanol for 1 h using an automated mixer, centrifuged at 4000 rpm for 5 min and supernatant transferred. The pellet was reextracted with 2 mL of 10% methanol with 0.2% formic acid followed by 2 mL of methanol. Supernatants were combined, evaporated to dryness and reconstituted in 500 µL of 95:5 (*v*:*v*) 0.2% formic acid/methanol. HPLC-radioprofiles for all samples were obtained using microplate solid scintillation counting. The column effluent was collected into Deepwell LumaPlate 96 (Perkin-Elmer, Waltham, MA, USA) in 15 s intervals for up to 48 min. Plates were dried in a Gene Vac EZ-2 evaporator (Genevac Inc, Gardiner, NY, USA) and counted on a Top Count NXT (Packard, Meriden, CT, USA) for up to 12 min per well. The chromatograms were reconstructed using ProFSA plus (Perkin-Elmer, Waltham, MA, USA). Each peak was expressed as %ROI (region of interest) and all integrated peaks together constituted a 100% ROI.

### 2.8. Metabolite Identification LC/MS

A Finnigan LCQ Advantage Max (Thermo Electron Corp., West Palm Beach, FL, USA) and a Micromass Q-TOF-2 (Waters Inc., Milford, MA, USA) was used in positive ion ESI mode. Samples were separated on a Supelco Discovery, HSF5, 3 μm, 15 cm × 4.5 mm HPLC column (Sigma-Aldrich, St. Louis, MO, USA) at ambient temperature and flow rate of 0.4 mL/min using a gradient elution using 0.2% formic acid and methanol. The LCQ was used at a spray voltage of 4.5 kV and collision energy of 35% and an isolation width of 2.0. The Q-TOF-2 was used at a capillary voltage of 2.0 kV and collision energy for full scan MSToF at 10 V and for MSMS at 30 V and a cone voltage of 35 V.

### 2.9. Calculations

Pharmacokinetic calculations were conducted using WinNonlin professional edition v5.2 (Pharsight Corporation, Cary, NC, USA). Calculation of percentage of radioactivity in red blood cells (RBC) was determined using the following equation: ((*Cb* − (*Cp* × (1 − H)))/*Cb*) × 100, where *Cp* = plasma concentration of radioactivity, *Cb* = blood concentration of radioactivity, and H = 0.45 for mice and H = 0.42 for dog [29].

## 3. Results

### 3.1. *In Vitro*

In order to assess the relative hydrolysis rates in liver and intestine, liver S9 fractions and small intestine homogenates were incubated with LY2334737 and the rate of gemcitabine release measured (Table 1). LY2334737 was slowly hydrolyzed (<35% in 6 h) in mouse, dog, monkey and human liver S9 fractions and to an even lesser degree (2% to 13%) in mouse, dog, monkey and human small intestine homogenates. This suggests that the extent of prodrug (LY2334737) hydrolysis in enterocytes is low, allowing significant absorption of intact prodrug. Enzyme kinetics for the hydrolysis of LY2334737 in liver S9 fractions and small intestine homogenates prepared from mice and human indicate that the enzyme(s) involved had relatively low affinity, with Km values of approximately 50 µM or higher (Table 2). The *K*m values in intestine and liver were similar (within 2-fold) within species suggesting a similar enzyme(s) could be responsible in both organs. The *V*max was substantially lower in intestine, leading to a lower intrinsic clearance in both species relative to liver, and is differentiated between tissues and is more prominent in mouse. The addition of loperamide, a known inhibitor of carboxylesterase 2 (CES2), to human S9 incubations caused an overall decrease in intrinsic clearance with increasing loperamide concentrations (Table 2). The loperamide inhibition pattern displays mixed-type inhibition with increase in *K*m and decrease in *V*max. Experiments using purified CES1A1 and CES2 showed that CES2 was responsible for the release of gemcitabine from LY2334737 with less than 1% turnover in CES1A1 incubation (Table 3). The *K*m and *V*max values reported here for human liver S9 fractions are very similar to the *K*m and *V*max values recently reported for LY2334737, using human recombinant CES2 [30], further supporting the role of CES2 in the clearance of LY2334737.

**Table 1:** Release of gemcitabine following the incubation of 100 µM LY2334737 in small intestine homogenates and 10 µM LY2334737 in liver S9 fractions for 6 h at 37 °C.

	Percent LY2334737 hydrolyzed to release gemcitabine			
	Mouse	Dog	Monkey	Human
Small intestine homogenate	9.5	13.1	2.1	3.0
Liver S9 fraction	17.2	7.4	34.2	34.9

**Table 2:** Enzyme kinetics for the release of gemcitabine from LY2334737 following incubation in small intestine homogenate, liver S9 fraction, and liver S9 fraction in the presence of loperamide.

	Mouse			Human		
	Km (µM)	Vmax (pmol/min/mg)	Intrinsic clearance (µL/min/mg)	Km (µM)	Vmax (pmol/min/mg)	Intrinsic clearance (µL/min/mg)
**Experiment 1**						
Small intestine homogenate	114.0	1.5	0.013	85.4	12.8	0.14
Liver S9 fraction	98.1	112.3	1.1	49.6	40.4	0.81
**Experiment 2**						
Liver S9	-	-	-	65.6	45.1	0.68
Liver S9 + 20 µM loperamide	-	-	-	132.9	32.2	0.24
Liver S9 + 100 µM loperamide	-	-	-	199.0	22.2	0.11

**Table 3:** Release of gemcitabine following the incubation of 200 µM LY2334737 with purified carboxyl esterase CES1A1 and CES2 for 2 h at 37 °C.

CES concentration (μg/mL)	Gemcitabine released (μM)
**CES1A1**	
1	0
10	0
100	0.9
**CES2**	
1	2.0
10	22.3
100	27.4

### 3.2. Pharmacokinetics

**Figure 2:**
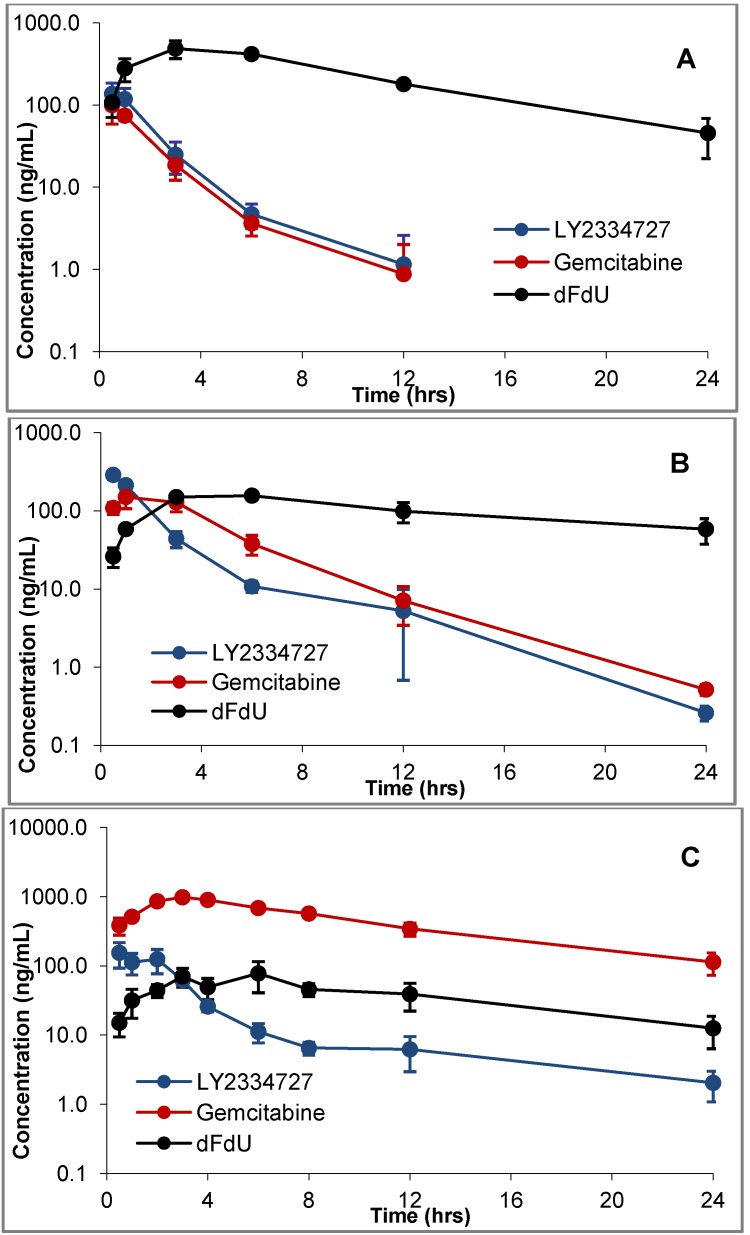
Plasma exposure profiles of LY2334737, gemcitabine, and dFdU following the administration of a single oral dose of LY2334737 at 4 mg/kg to CD-1 mice (**A**), 1 mg/kg to Beagle dogs (**B**) and 10 mg/kg to Sprague-Dawley rats (**C**). Mean and standard deviation based on *n* = 3.

The plasma exposure profiles in mice, rats and dogs following a single oral dose of LY2334737 are shown in Figure 2. LY2334737 was rapidly absorbed with a *T*_max_ of approximately 0.5 h in all three species, and was hydrolyzed to release gemcitabine. Gemcitabine exposures were quantifiable up to 12 h in mice and up to 24 h in both rats and dogs, which is a contrasting difference compared to exposure profiles following IV administration of gemcitabine [31]. Gemcitabine was subsequently metabolized by cytidine deaminase to its inactive metabolite dFdU, which is seen circulating at high concentrations in mice and dogs but not in rats.

A comparison of the plasma exposures following a single oral dose of LY2334737 and a single oral dose of gemcitabine to mice and dogs are summarized in Table 4. In mice, the administration of gemcitabine resulted in a substantial first-pass deamination leading to the rapid formation and accumulation of dFdU compared to the administration of LY2334737, as evidenced by the large differences between the metabolite ratios observed following the administration of the two compounds. The same trend was observed in dogs also; however, the difference was less pronounced. Higher metabolite ratios suggest more extensive deamination of gemcitabine resulting in the formation of dFdU. Overall, the administration of LY2334737 resulted in a lower metabolite ratio compared to the administration of gemcitabine, indicating less extensive deamination of gemcitabine. A gender effect was observed in mice where the females had higher exposures of dFdU and correspondingly higher metabolite ratios compared to males.

**Table 4:** Comparison of plasma exposure in male CD-1 mice and female Beagle dogs following a single oral dose of gemcitabine (dFdC) and a single oral dose of LY2334737.

	Mouse (AUC_0–24_ ng·h/mL)				Dog (AUC_0–24_ ng·h/mL)			
	2 mg/kg gemcitabine		4 mg/kg ^a^ LY2334737		0.5 mg/kg gemcitabine		1 mg/kg ^b^ LY2334737	
	male	female	male	female	male	female	male	female
Gemcitabine (dFdC)	92.7	95.5	207	189	808	1,090	645	799
dFdU	5,170	14,300	2,580	5,360	4,928	4,616	2,310	2,400
LY2334737	na	na	324	276	na	na	656	617
Metabolite Ratio^c^	55.8	149.7	12.5	28.4	6.1	4.2	3.6	3.0

^a^ equivalent to 2.7 mg/kg gemcitabine; ^b^ equivalent to 0.675 mg/kg gemcitabine; ^c^ dFdU/dFdC.

Exposure profiles following the administration of a single oral dose of radiolabelled LY2334737 to mice and dogs were similar to Figure 2 and the resulting pharmacokinetic parameters are summarized in Table 5. The time *vs* concentration profiles were similar to those observed following dosing unlabelled compound (Figure 2). The mice had short terminal half-lives for both LY2334737 and gemcitabine (1.2 to 1.4 h) while they were longer in the dogs (7 to 8 h). The metabolite dFdU and total radioactivity had long half-lives and were circulating in plasma at higher levels and for a longer period of time than LY2334737 and gemcitabine in both species. Overall, dFdU was the major circulating metabolite and accounted for approximately 70% of the total circulating plasma radioactivity in mice and approximately 58% in dogs. The absolute oral bioavailability of LY2334737 was 53% in dogs. The elimination half-life of dFdU was comparable to that of total radioequivalents suggesting that the elimination of the metabolite dFdU contributes to the longer terminal half-life of total radioactivity in plasma.

**Table 5:** Summary pharmacokinetic parameters of LY2334737, gemcitabine, and dFdU following a single oral dose of ^14^C**-**LY2334737 in male CD-1 mice and a single oral and IV dose ^14^C**-**LY2334737 in female Beagle dogs.

Parameter		Radioactivity in Plasma	LY2334737 in Plasma	dFdU ^a^ in Plasma	dFdC ^a^ in Plasma
**Mouse** (10 mg/kg oral, *n* = 4 composite sampling)					
AUC_0–t_ (ng equiv·h/mL or ng·h/mL)		24,700	822	17,244	741
*C*_max_ (ng equiv/mL or ng/mL)		4,350	294	2,741	280
*T*_½_ (h)		14.9	1.2	NC ^b^	1.4
*T*_max_ (h)		2.0	1.0	2.0	0.5
**Dog** (5 mg/kg oral, *n* = 3)					
AUC_0–t_ (ng equiv·h/mL or ng·h/mL)		57,200 ± 11,300	2,610 ± 495	32,720 ± 7,296	3,906 ± 1,289
*C*_max_ (ng equiv/mL or ng/mL)		4,950 ± 1,330	1,490 ± 305	1,548 ± 209	795 ± 330
*T*_½_ (h)		11.9 ± 2.8	7.7 ± 3.0	12.1 ± 2.8	6.6 ± 1.8
*T*_max_ (h)		1.0 ± 0.0	0.7 ± 0.3	5.3 ± 2.3	1.0 ± 0.0
**Dog** (5 mg/kg IV, *n* = 3)					
AUC_0–t_ (ng equiv·h/mL or ng·h/mL)		56,000 ± 8,760	4,890 ± 608	32,720 ± 4,348	3,654 ± 1,027
*C*_0_ or *C*_max_ (ng equiv/mL or ng/mL)		11,300 ± 1,810	7,700 ± 1,710	1,739 ± 196	748 ± 189
*T*_½_ (h)		14.1 ± 2.5	6.5 ±	14.3 ± 3.2	7.3 ± 2.1
*T*_max_ (h)		na	na	3.3 ± 1.2	0.5 ± 0.0

^a^ expressed as LY2334737 equivalents; ^b^ due to insufficient data points to define the terminal phase; Abbreviations: AUC_0–t_ = area under the plasma concentration-time curve from time = 0 to last quantifiable time point, *C*_max_ = maximal plasma concentration, NC = not calculated , *T*_½_ = terminal elimination half-life, and *T*_max_ = time to maximal plasma concentration.

### 3.3. Excretion

**Figure 3:**
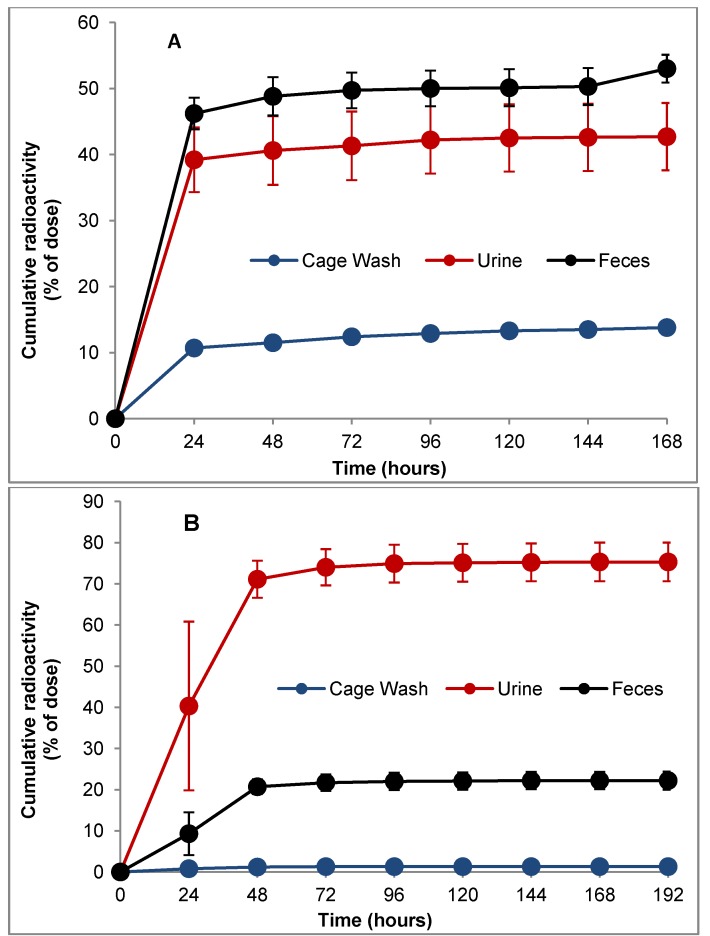
Cumulative percent recovery of radioactivity: from CD-1 mice (**A**) following a single oral dose of 10 mg/kg ^14^C-LY2334737; and Beagle dogs (**B**) following a single oral dose of 5 mg/kg ^14^C-LY2334737.

Cumulative excretion profiles following administration of a single oral dose of ^14^C-LY2334737 to mice and dogs are shown in Figure 3. In both mice and dogs the main route of excretion of total radiocarbon was via the feces (53.0% and 75.3%, respectively) followed by the urine (42.7% and 22.2%, respectively). Greater than 90% of the total radioactivity was recovered within the first 24 h in mice and within the first 48 h in the dogs. The mean total recovery of radioactivity was greater than 98%. The excretion profiles from the dogs administered a single IV dose of ^14^C-LY2334737 was almost identical to oral dosing. Overall, the mass balance data indicate that both renal and biliary excretion was involved in the clearance of LY2334737 and related radioactivity in both mice and dogs.

### 3.4. RBC Partitioning and Protein Binding

Approximately 37% to 42% of LY2334737-related radioactivity partitioned into red blood cells in mice and approximately 43% to 46% in dogs, indicating no preferential uptake. LY2334737 demonstrated low protein binding in mice and dogs (70.4% bound in mouse and 73.1% bound in dogs) as determined by ultracentrifugation. Gemcitabine has no discernible protein binding as reported previously [31].

### 3.5. Metabolism

Metabolite profiles were examined from samples collected following the administration of a single dose of ^14^C-LY2334737. The major circulating metabolite in both mice and dogs was dFdU, followed by LY2334737 and gemcitabine (dFdC) (Figure 1). Additional minor metabolites included glucuronides of LY2334737, gemcitabine and dFdU in dog plasma and oxidative (+O and +2O–2H) metabolites of LY2334737 in mouse plasma. Overall, greater than 85% of the total plasma radioactivity was identified in mice and 88% in dogs.

A summary of the metabolite profiles obtained following a single dose of ^14^C-LY2334737 to mice and dogs are shown in Table 6. The major entity in mouse and dog urine was dFdU accounting for approximately 23% and 39% of the total dose, respectively, followed by dFdU glucuronide and LY2334737. The major entity in mouse and dog feces was LY2334737 accounting for approximately 24% and 12% of the total dose, respectively, followed by dFdU. Other less abundant metabolites identified included multiple oxidative metabolites of LY2334737 and glucuronides of LY2334737, dFdU, and gemcitabine. The qualitative profiles obtained following IV dosing to dogs were comparable to those following oral dosing.

**Table 6:** Summary of metabolites (percentage of dose) in the urine and feces of CD-1 mice and Beagle dogs following a single 10 mg/kg and 5 mg/kg oral dose of ^14^C-LY2334737, respectively.

Metabolite	Total percentage (%) of dose			
	Mouse Urine	Dog Urine	Mouse Feces	Dog Feces
	0 to 96 h	0 to 72 h	0 to 96 h	0 to 72 h
LY2334737	4.0	12.4	23.7	11.7
LY2334737-2H, +O, or +2O–2H	2.4	0.7	4.4	2.0
Gemcitabine (dFdC)	2.3	3.1	nd	nd
dFdU	22.6	39.1	13.4	1.4
LY2334737 + glucuronide	nc^a^	6.3	nd	nd
dFdC + glucuronide	0.7	1.5	nd	0.7
dFdU + glucuronide	7.4	6.7	0.3	0.4
Amount Excreted ^b^	42.2	74.0	49.7	21.7

^a^ Peaks detected but not calculated due to co-elution with an adjacent peak; ^b^ Percent of dose excreted during specified timeframe; nd = not detected.

## 4. Discussion and Conclusions

Oral administration of gemcitabine and similar oncolytics could be advantageous in allowing novel dose schedules. However, previous studies have demonstrated that oral delivery of gemcitabine is limited by its low oral bioavailability and potential intestinal toxicity [21]. The current data describe the preclinical dispositional properties of LY2334737, an amide prodrug of gemcitabine that was developed to achieve good systemic exposure of gemcitabine via improved intestinal permeability and bioavailability while avoiding intestinal toxicity.

Historical data from preclinical mouse and dog studies show that gemcitabine is readily deaminated by cytidine deaminase to its inactive metabolite dFdU. The metabolite dFdU circulates at higher concentrations than parent drug in mice and dogs; however, this is not the case in rats [31]. The low conversion to dFdU in rats is attributed to lower levels of cytidine deaminase [31], resulting in a significant alteration in gemcitabine clearance. Clinical data also show that gemcitabine is rapidly deaminated following IV administration to patients, with clearance patterns more similar to mice than rats [32]. Therefore, mice were selected as the rodent species, and not rats, during preclinical development of LY2334737.

Systemic exposure of gemcitabine is contingent upon intact absorption of LY2334737 bypassing intestinal deamination, requiring LY2334737 to be relatively resistant to activation during the absorption process. In vitro studies were conducted to assess the rate of hydrolysis of LY2334737 in liver and intestine across species. In all species tested, the rate of *in vitro* hydrolysis in intestinal preparations was low, with less than 14% hydrolysis over 6-hour incubation. This slow conversion was predicted to result in low intestinal activation *in vivo* (Table 1, Table 2). In human intestinal and hepatic preparations, intrinsic clearance values for LY2334737 were similar, suggesting that LY2334737 can be activated in both organs during first-pass, albeit at a slow rate. Therefore, further experiments were conducted to help elucidate the potential enzymes involved in activation of this prodrug.

Because LY2334737 was hydrolyzed by intestinal enzymes, it was hypothesized that carboxylesterase 2 (CES2) could be involved. Of the two predominant human CES isoforms, only CES2 is highly expressed in both liver and intestine, and the expression of CES1 has been shown to be liver-specific [33,34]. They seem to have broad and overlapping substrate specificities and are known to hydrolyze a variety of drug esters, amides, carbamates, and similar structures [35]. Loperamide, a selective CES2 inhibitor, inhibited LY2334737 hydrolysis in a concentration-dependent manner, further implicating CES2 in human activation of LY2334737. To directly confirm the involvement of CES2, LY2334737 was incubated with expressed CES1 and CES2 enzymes. Hydrolysis was only observed with CES2, with the hydrolysis rate increasing linearly with enzyme concentration. Less than 1% of the initial LY2334737 was converted to gemcitabine by CES1 during 2 h incubation. Collectively, these results indicate that the activation of LY2334737 in humans is likely to be carried out by CES2. Additional data implicating CES2 in activating LY2234737 have been recently reported [30]. We hypothesized that the slow *in vitro* hydrolysis of LY2334737 in intestine and liver would result in a significant fraction of an oral dose to be absorbed intact along with a relatively slow systemic release *in vivo*.

Data presented here confirm that oral administration of LY2334737, to preclinical species results in a prolonged systemic exposure of gemcitabine and supports the avoidance of first pass clearance [25], compared to the extremely rapid clearance of gemcitabine observed in preclinical species following administration of gemcitabine intravenously [31]. LY2334737 was rapidly absorbed intact following oral dosing and eliminated in feces and urine within 24 h as demonstrated from the radiolabeled studies. Comparison of the excretion profiles following oral and IV administration support the involvement of biliary excretion in addition to renal excretion. In contrast, renal excretion was the predominant route of elimination of gemcitabine and related radioactivity following IV administration of gemcitabine to mice, rats, and dogs [31] as well as in patients (unpublished data). Therefore, administration of LY2334737 demonstrates diversity in its clearance routes and could be advantageous from a clinical perspective.

The predominant pathway of LY2334737 metabolism is via hydrolysis of the amide linkage releasing valproic acid and gemcitabine. Oral administration of LY2334737 displays formation-rate limited kinetics and the resulting pharmacokinetic profiles tend to mimic a systemic sustained release of gemcitabine. Once gemcitabine is released, it is available to be activated and incorporated in to the tumor DNA and the remainder is metabolized and eliminated in urine and feces. *In vitro* studies as well as human colon and lung tumor xenograft studies have shown that metronomic low doses of LY2334737 is efficacious and well-tolerated [36]. Therefore, the ability to almost continuously deliver “smaller-doses” of gemcitabine, as demonstrated in the preclinical studies, may prove advantageous to treating select tumor types that respond to a metronomic dosing approach.

Overall, oral administration of LY2334737 resulted in successful delivery of gemcitabine to systemic circulation, with reduced liability of gastrointestinal toxicity typically observed following the oral administration of gemcitabine. The *in vitro* hydrolysis rates were consistent with low intestinal activation and formation rate limited pharmacokinetics.
